# Supplementary figures and images for: Cell-autonomous role of leucine-rich repeat kinase in the protection of dopaminergic neuron survival
Source: eLife. 2024 Jun 10;12:RP92673. doi: 10.7554/eLife.92673 (PMC11164531; doi:10.7554/eLife.92673)

Figure 1-I  
LRRK1 WB

control  
*Lrrk1*  $\Delta/\Delta$

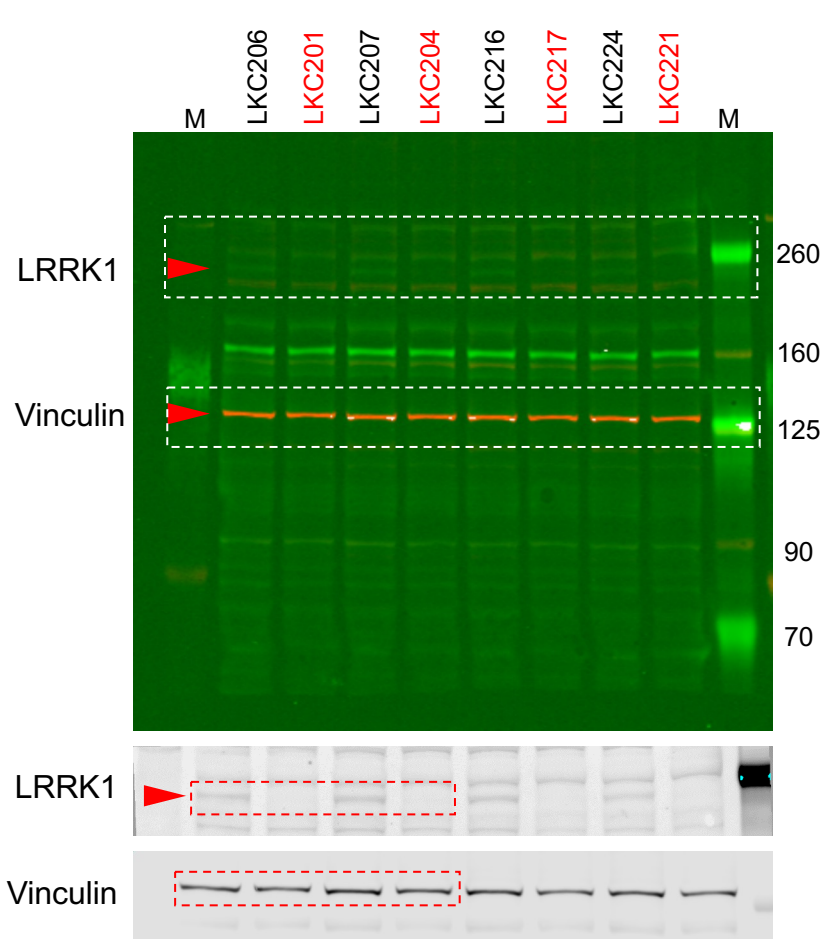

Figure 1-I  
LRRK2 WB

control  
*Lrrk2*  $\Delta/\Delta$

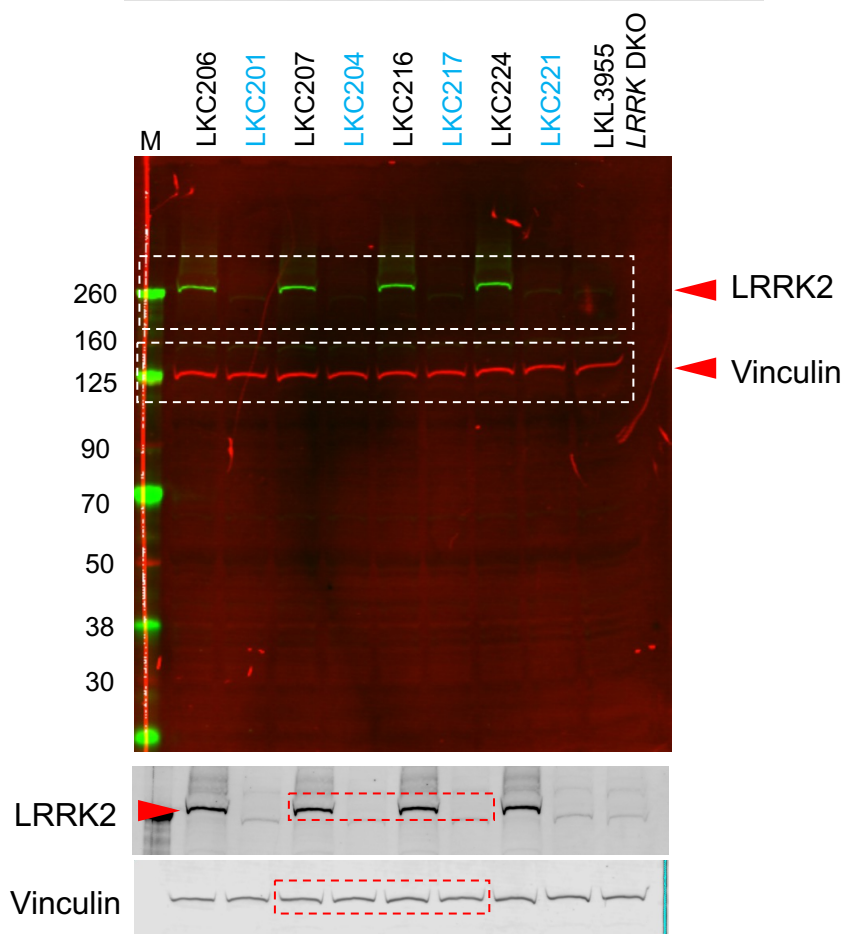

Supplement: Figure 1—source data 1. [file elife-92673-fig1-data1.zip › Kang_Figure 1 - source data 1/Kang_Figure 1 - source data 1 - uncropped blot with relevant bands labelled.pdf]

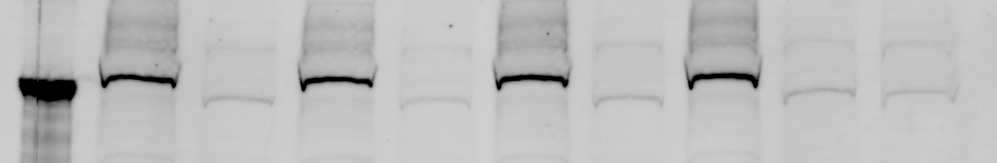

Supplement: Figure 1—source data 1. [file elife-92673-fig1-data1.zip › Kang_Figure 1 - source data 1/Kang_Figure 1 - source data 1 - original file of full unedited blot/Figure 1 - source data 1 -Fig1I-PD-18-2-LRRK2-KO-CTX-LRRK2-High.tif]

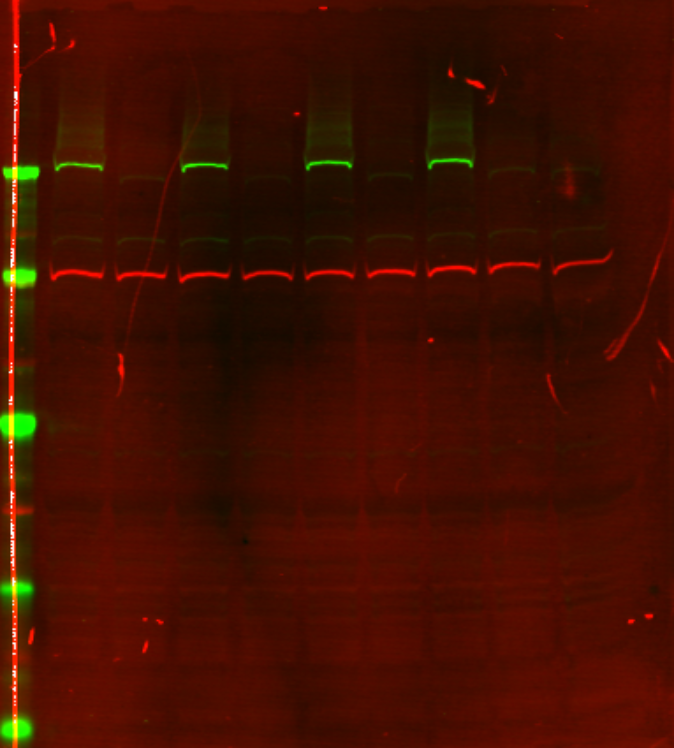

Supplement: Figure 1—source data 1. [file elife-92673-fig1-data1.zip › Kang_Figure 1 - source data 1/Kang_Figure 1 - source data 1 - original file of full unedited blot/Figure 1 - source data 1 - Fig1I-PD-18-1-LRRK2-KO-CTX-LRRK2-Vinculin-low.tif]

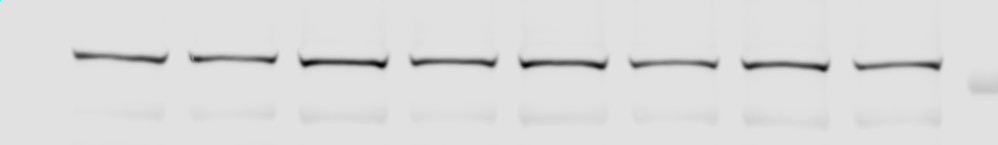

Supplement: Figure 1—source data 1. [file elife-92673-fig1-data1.zip › Kang_Figure 1 - source data 1/Kang_Figure 1 - source data 1 - original file of full unedited blot/Figure 1 - source data 1 -Fig1I-PD23-1-LRRK1-KO-CTX-LRRK1-Alomone-Vinculin-High.tif]

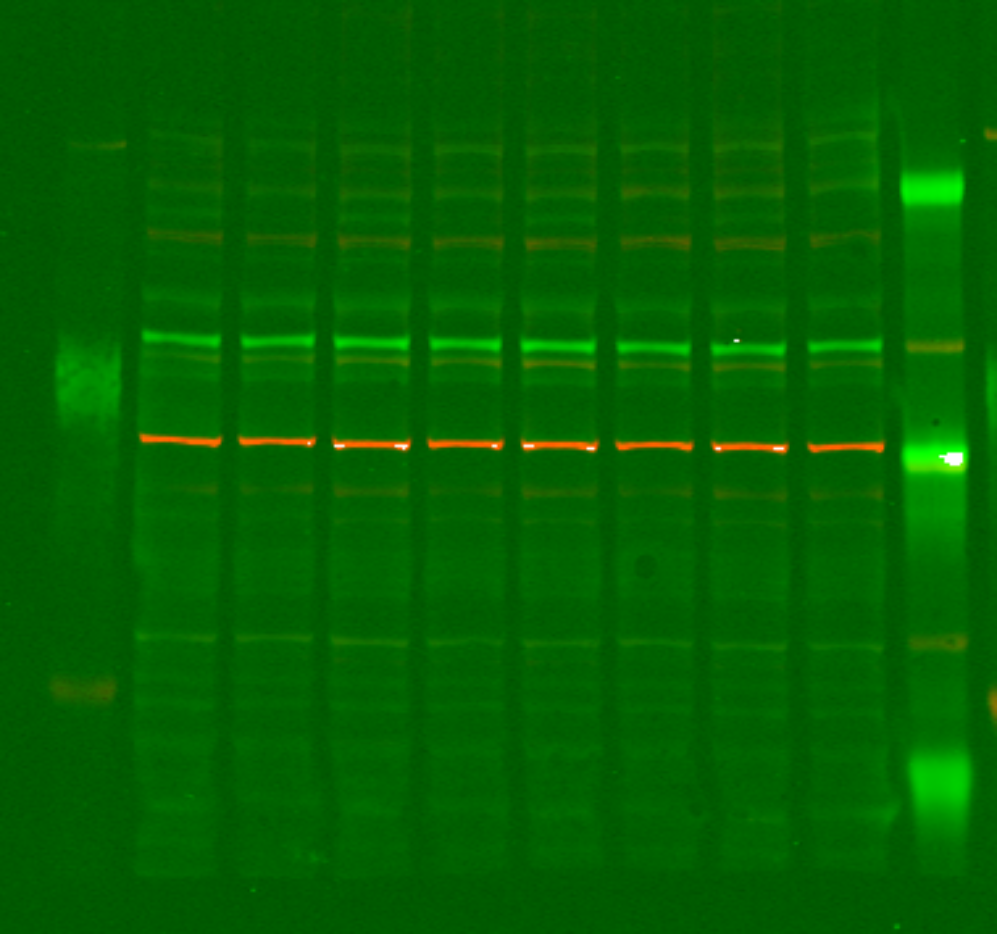

Supplement: Figure 1—source data 1. [file elife-92673-fig1-data1.zip › Kang_Figure 1 - source data 1/Kang_Figure 1 - source data 1 - original file of full unedited blot/Figure 1 - source data 1 -Fig1I-PD23-1-LRRK1-KO-CTX-LRRK1-Alomone-Low.tif]

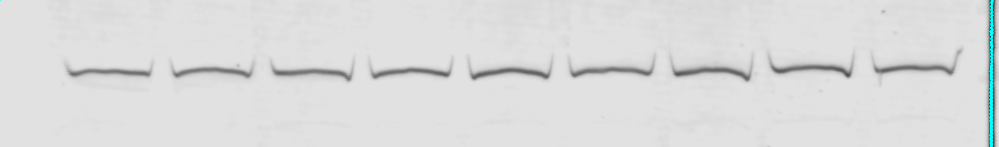

Supplement: Figure 1—source data 1. [file elife-92673-fig1-data1.zip › Kang_Figure 1 - source data 1/Kang_Figure 1 - source data 1 - original file of full unedited blot/Figure 1 - source data 1 -Fig1I-PD-18-2-LRRK2-KO-CTX-LRRK2-Vinculin-High.tif]

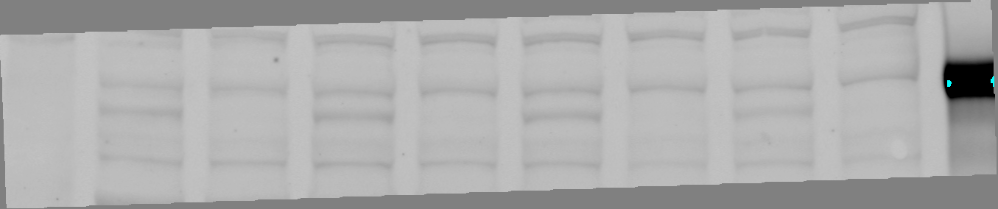

Supplement: Figure 1—source data 1. [file elife-92673-fig1-data1.zip › Kang_Figure 1 - source data 1/Kang_Figure 1 - source data 1 - original file of full unedited blot/Figure 1 - source data 1 -Fig1I-PD23-1-LRRK1-KO-CTX-LRRK1-Alomone-High.tif]
