# Supplementary material for: Cell-autonomous role of leucine-rich repeat kinase in the protection of dopaminergic neuron survival
Source: eLife. 2024 Jun 10;12:RP92673. doi: 10.7554/eLife.92673 (PMC11164531; doi:10.7554/eLife.92673)
Supplement: Supplementary file 1. [file elife-92673-supp1.docx]

**Supplementary file 1. Generation of the *Lrrk1* targeting vector**

**1)** The *left middle* arm was amplified by PCR from mouse BAC DNA (Clone #: RP23-213J23, BACPAC Resources Center) using primers P3 and P4. The resulting PCR product (2,079 bp) containing part of *Lrrk1* intron 26, exon 27, and part of intron 27 was subcloned into the pGEM-T Vector (Promega, Cat#; A1360) by TA ligation to generate pLRRK1#1 (pLM1).

P3: 5’CCAGTCACTTCTCCACCTCAGGGAAAATGG (30 bp)

P4: 5’CCTTGT*GGTACC*CGGACCTTCTATCACCTTTATCC (35 bp): *Kpn*I (*GGTACC*) is an endogenous restriction site.

*
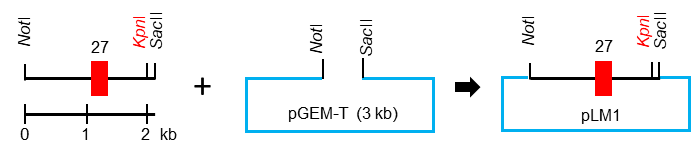
*

**2)** The *right middle* arm was amplified from mouse BAC DNA (Clone #: RP23-213J23) using primers P5 and P6. The resulting PCR product (3,403 bp) containing part of *Lrrk1* intron 27, exon 28, intron 28, exon 29, and part of intron 29 was subcloned into the pGEM-T Vector by TA ligation to generate pLRRK1#2 (pLM2).

P5: 5’GGTCCG*GGTACC*ACAAGGTGCTGGTTAAGTGCC (33 bp): *Kpn*I (*GGTACC*) is an endogenous restriction site.

P6: 5’AGCAGACCTCTTGCCTTCTACTACTGACTG (30 bp)


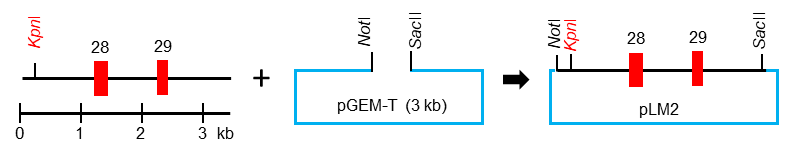


**3)** pLRRK1#1 (pLM1) was digested with *Not*I and *Kpn*I, then subcloned into the *Not*I and *Kpn*I sites of pLRRK1#2 (pLM2) to generate the pLRRK1#3 (pLM3) plasmid which contains the *middle* homologous region.


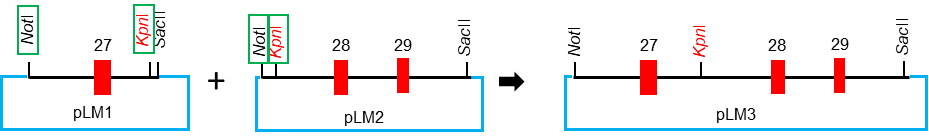


**4)** The middle homologous region was released from pLRRK1#3 (pLM3) using *Not*I and *Sac*II followed by Klenow to blunt the sticky ends, which was then subcloned into the *Sma*I site of pGKneoF2L2DTA (addgene: #13445) to generate pLRRK1#4 (pLM4).

Green arrowhead: *loxP* site, gray circle: *FRT* site

**5)** The left arm was amplified from mouse BAC DNA (Clone: RP23-213J23) using primers P1 and P2. The resulting PCR product (2,016 bp) containing part of *Lrrk1* intron 25, exon 26, and part of intron 26 was digested with *Sac*II and *Not*I, and was then subcloned into the *Sac*II and *Not*I sites of pGKneoF2L2DTA (addgene: #13445) to generate pLRRK1#5 (pLM5).

P1: 5’gacat*CCGCGG*CACCATGTGAGTGGCAGCTGTGGTGAGAAC (41 bp). *Sac*II (*CCGCGG*) is an exogenous site.

P2: 5’gacat*GCGGCCGCAAGCTT*TTTAATAGCCGTTCTTTCTTAGAGAAGGCAG (50 bp). *Not*I (*GCGGCCGC*) and *Hind*III (*AAGCTT*) are exogenous sites.

**6)** The right arm was amplified from mouse BAC DNA (Clone: RP23-213J23) using primers P7 and P8. The resulting PCR product (3,131 bp) containing part of *Lrrk1* intron 29, exon 30, and part of intron 30 was digested with *Sal*I and *Hind*III, and was then subcloned into the *Sal*I and *Hind*III sites of pGKneoF2L2DTA (addgene: #13445) to generate pLRRK1#6 (pLM6).

P7: 5’gacat*GTCGAC*GGATCCGTAGGGAAGACCCACTAGGAGGAAGAAAG (46 bp). *Sal*I (*GTCGAC*) is an exogenous site.

P8: 5’gacat*AAGCTT*TGGTACCTTTCTAAAGGCAGCATTTTGCTTGC (43 bp). *Hind*III (*AAGCTT*) is an exogenous site.

**7)** pLRRK1#5 (pLM5) was digested with *Sac*II and *Not*I, and was then subcloned into the *Sac*II and *Not*I sites of pLRRK1#6 (pLM6) to generate pLRRK1#7 (pLM7).

**8)** The middle homologous region was released from pLRRK1#4 by *Not*I and *Sal*I and was then subcloned into the *Not*I and *Sal*I sites of pLRRK1#7 (pLM7) to generate the final targeting vector pLRRK1#8 (pLM8), which was linearized with *Xho*I before electroporation into ES cells.

Restriction sites in black: from vectors

Restriction sites in red: *Lrrk1* endogenous sites

Restriction sites in blue: introduced by the primers
