# Supplementary material for: Cell-autonomous role of leucine-rich repeat kinase in the protection of dopaminergic neuron survival
Source: eLife. 2024 Jun 10;12:RP92673. doi: 10.7554/eLife.92673 (PMC11164531; doi:10.7554/eLife.92673)
Supplement: Supplementary file 2. [file elife-92673-supp2.docx]

**Supplementary file 2. Generation of the *Lrrk2* targeting vector**

**1)** The left homologous region was amplified from mouse BAC DNA (Clone# RP23-526-A2, BACPAC Resources Center) using primers P9 and P10. The resulting PCR product (2,579 bp) was digested with *EcoR*I, which is an endogenous restriction site upstream of the *Lrrk2* promoter, and was then subcloned into the *EcoR*I and *Sma*I sites of pBSK (+) to generate pLRRK2#1 (2,526 bp + 2,961 bp = 5,487 bp).

P9: 5’ GAACACACAAGGCTATGGCTATTGTC (26 bp)

P10: 5’ GTAGGACTATCATCCACCTGTAGGACTCC (29 bp)


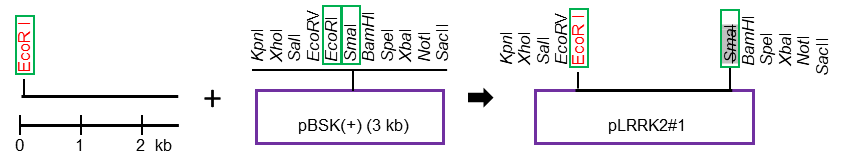


**2)** Oligoes (P39 and P40) were annealed together to generate a double stranded polylinker (*BamH*I-*loxP*-*Nhe*I-*Spe*I), which was then subcloned into the *BamH*I and *Spe*I sites of pLRRK2#1 to generate pLRRK2#2 (5,487 bp + 50 bp = 5,537 bp).


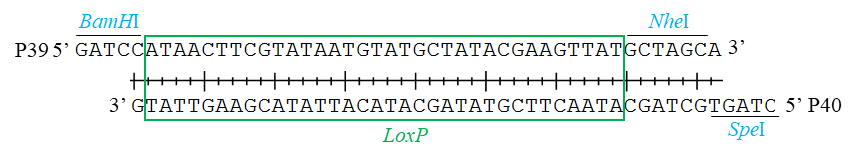


P39: 5’ GATCCATAACTTCGTATAATGTATGCTATACGAAGTTATGCTAGCA (46 bp).
*BamH*I (G'GATCC), *loxP* top strand (ATAACTTCGTATAATGTATGCTATACGAAGTTAT), *Nhe*I (G'CTAGC).

P40: 5’ CTAGTGCTAGCATAACTTCGTATAGCATACATTATACGAAGTTATG (46 bp).
*Spe*I (A'CTAGT), *Nhe*I (GCTAGC), *loxP* bottom strand (ATAACTTCGTATAGCATACATTATACGAAGTTAT).


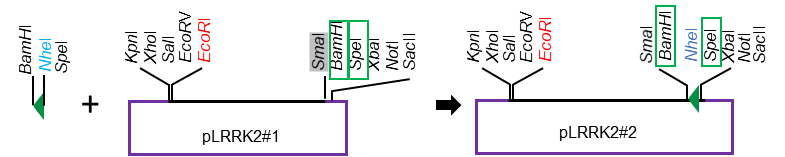


Green arrowhead: *loxP* site

**3)** The middle arm was amplified from mouse BAC DNA (Clone# RP23-526-A2) using primers P11 (upstream of the *Lrrk2* promoter) and P12 (intron 2). The resulting PCR product (2,990 bp) was digested with *Xba*I and *Not*I and was then subcloned into the *Xba*I and *Not*I of pGEM-T Vector (Promega, Cat#: A1360) to generate pLRRK2#3 (2,990 bp + 3,000 bp = 5,990 bp). *Xba*I, *Not*I, and *Nhe*I sites are exogenous sequences introduced into PCR primers.


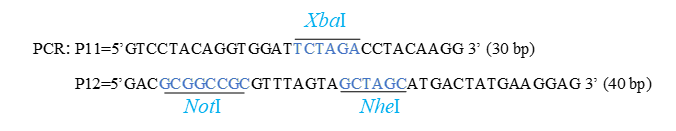


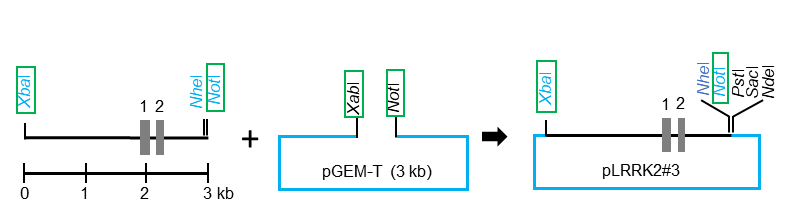


**4)** pLRRK2#3 was digested with *Xba*I and *Not*I, and the insert was then subcloned into the *Xba*I and *Not*I sites of pLRRK2#2, which contains the left homologous arm and the *loxP* sequence, to generate pLRRK2#4 (2,958 bp + 5,490 bp = 8,448 bp).


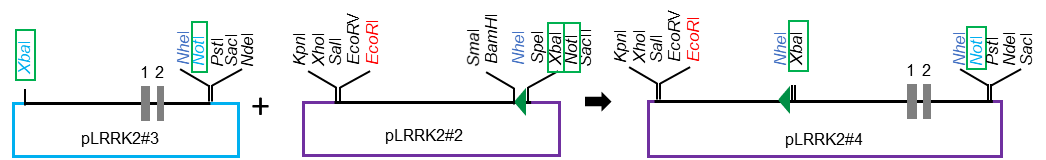


**5)** The right arm was amplified from mouse BAC DNA (Clone# RP23-526-A2) using primers P13 and P14. The resulting 3,503 bp PCR fragment containing *Lrrk2* intron 1, exon 2, and intron 2, was subcloned into the *EcoR*V site of pBSK(+) to generate pLRRK2#5 (3,503 bp + 2,961 bp = 6,464 bp). **Check direction!!!**

PCR: P13=5’GCACTTGAGTCTTAATCTTGGGCAC 3’ (25 bp)

P14=5’CATTCGAGCAGCTAAGCCTGTAATC 3’ (25 bp)


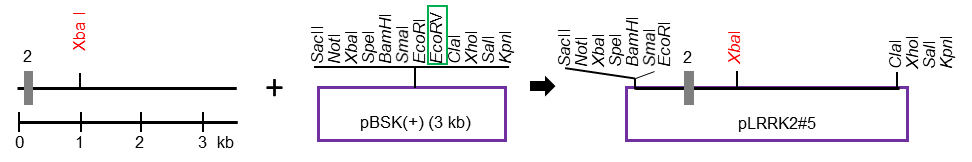


**6)** pLRRK2#5 was digested with *BamH*I and then treated with Klenow to blunt the overhang followed by *Cla*I digestion. The digested fragment was subcloned into the *EcoR*V and *Cla*I sites of the pSoriano vector (*PGKneolox2DTA*, addgene 13443) to generate pLRRK2#6 (3,547 bp + 6,307 bp = 9,854 bp).

**7)** The LFNT-tk/pBS plasmid (a gift from S. Tonegawa) was digested with *Sac*II (followed by Klenow) and *Not*I (followed by *Ssp*I) to release the “*loxP-FRT-Pgk-neo-loxP-FRT*” fragment (2,928 bp), which was then subcloned into the *Xba*I (followed by Klenow) and *Not*I sites of pLRRK2#6 to generate pLRRK2#7 (2,928 bp + 7,051 bp = 9,979 bp).

Green arrowhead: *loxP* site, gray circle: *FRT* site

**8)** pLRRK2#4 was digested with *EcoR*V and *Not*I to release the fragment containing the left arm-*loxP*-middle arm, which was then subcloned into the *Ale*I and *Not*I sites of pLRRK2#7 to obtain pLRRK2#8, the *Lrrk2* targeting vector (15,485 bp). The linearized targeting vector (with *Ahd*I) was electroporated into ES cells.

Restriction sites in black: from vectors

Restriction sites in red: *Lrrk2* endogenous sites

Restriction sites in blue: introduced by the primers
